# Effects of Microcystin-LR on Metabolic Functions and Structure Succession of Sediment Bacterial Community under Anaerobic Conditions

**DOI:** 10.3390/toxins12030183

**Published:** 2020-03-15

**Authors:** Qin Ding, Kaiyan Liu, Zhiquan Song, Rongli Sun, Juan Zhang, Lihong Yin, Yuepu Pu

**Affiliations:** 1 Key Laboratory of Environmental Medicine Engineering, Ministry of Education of China, School of Public Health, Southeast University, Nanjing 210009, China; dingqin@seu.edu.cn (Q.D.);; 2 Department of Civil and Environmental Engineering, University of California, Irvine, CA 92697, USA

**Keywords:** microcystin, bacterial community, metabolic profile, high throughput analysis, anaerobic condition

## Abstract

Microcystins (MCs), which are produced by harmful cyanobacteria blooms, pose a serious threat to environmental health. However, the effect of MCs on the bacterial community under anaerobic conditions is still unclear. This study examined the dynamic changes of MC-degrading capacity, metabolic activity, and structure of the bacterial community in lake sediment repeatedly treated with 1 mg/L microcystin-LR (MC-LR) under anaerobic conditions. The results showed that the MC-degrading capacity of the bacterial community was increased nearly three-fold with increased treatment frequency. However, the metabolic profile behaved in exactly opposite trend, in which the overall carbon metabolic activity was inhibited by repeated toxin addition. Microbial diversity was suppressed by the first addition of MC-LR and then gradually recovered. The 16S amplicon sequencing showed that the dominant genera were changed from *Exiguobacterium* and *Acinetobacter* to *Prosthecobacter*, *Dechloromonas*, and *Agrobacterium*. Furthermore, the increase in the relative abundance of *Dechloromonas*, *Pseudomonas*, *Hydrogenophaga*, and *Agrobacterium* was positively correlated with the MC-LR treatment times. This indicates that they might be responsible for MC degradation under anaerobic conditions. Our findings reveal the relationship between MC-LR and the sediment bacterial community under anaerobic conditions and indicate that anaerobic biodegradation is an effective and promising method to remediate MCs pollution.

## 1. Introduction

The heavy occurrence of harmful cyanobacterial blooms has become a global environmental problem due to producing a series of cyanotoxins such as microcystins (MCs), nodularins, and cylindrospermopsins [1,2]. MCs, which account for the highest proportion of all cyanotoxins, are suggested to be involved in various biological functions, including resisting oxidative stress, nutrients acquisition and programmed cell death [3,4,5,6]. The MCs, retained intracellularly by intact cells, are released to the extracellular environment due to cell death or injury of structural integrity [7]. Moreover, MCs have been associated with liver damage, reproductive toxicity, and neurotoxicity, based on experimental data and epidemiological investigations [8,9]. Some variants of microcystin (MC), with a stabilized structure and severe toxicity, have often been detected in freshwater, sediment, and aquatic products, which not only disturb the internal balance of the aquatic ecology but also pose a potential threat to public health [10]. Microcystin-LR (MC-LR) is considered to be one of the most toxic variants, classified by International Agency for Research on Cancer (IARC) as a possible human carcinogen [10,11,12].

MCs exist not only in freshwater but are also detectable in sediment. MCs could be deposited in the sediment through various pathways, including the lysis of cyanobacterial cells immobilized in sediments, the sedimentation of natural organic matters on whose surface MCs are adsorbed, and the sinking MC-containing fecal matters grazed by aquatic animals [13,14]. Some MCs producers, e.g., *Microcystis* sp., can thrive in the benthic environment [15]. For these reasons, the concentrations of MCs in sediment are relatively higher than those in freshwater. Concentrations of MCs were reported to be between 0.06–0.78 mg/kg in the sediments of Lake Yangebup, Western Australia, during August and December, 2010 [16]. Previous researchers have characterized the changes in bacterial communities’ composition in freshwater during the outbreak of cyanobacterial blooms and considered that the microbial structure might be affected by the seasonal production of MCs and secondary metabolites of cyanobacteria [17,18,19]. The microbial content in the sediment was considered to be relatively higher than that of the water. However, the relationship between MCs and the bacterial community in sediments has been rarely studied. Furthermore, the characteristics of the bacterial community are important indicators of soil ecosystem health, and many functional microbes exist in the sediment [10]. Chen et al. found higher concentrations and degradation of MCs in lake sediment than in lake water, which suggested that the bacterial community in sediment may play a critical role in MCs degradation to maintain a relatively low level of cyanotoxin [20,21]. Therefore, it is necessary to investigate the relationship between MCs and the bacterial community in sediments. 

Due to the thick coating of cyanobacteria on the surface and the turbidity of the waterbody, less oxygen in the air can dissolve into deep water. The photosynthesis of cyanobacteria and plants is mostly weakened without sufficient sunlight. Contrastingly, the subsequent growth of zooplankton increases the consumption of oxygen in water. Dissolved oxygen is further consumed by aerobic microorganisms to decompose organic matter, especially during summer [22]. In this situation, dissolved oxygen in deep water and sediment would be at a low level. A large proportion of MCs exist in hypoxic and/or anaerobic environments, but most of the studies on MCs were carried out under aerobic conditions [3,10]. In addition, methanogenesis and denitrification may often co-occur with biodegradation [23]. Conrad et al. also indicated that organic matter was metabolized through anaerobic methods, mainly in the sediments of freshwater lakes [24]. The bacterial communities of environmental samples can effectively degrade MCs under anaerobic conditions, and it is a non-negligible method for the elimination of organic pollutants [25,26]. Therefore, more attention should be paid to the biological properties of the bacterial community and biodegradation of MCs under anaerobic conditions.

The high proportion of the active bacterial population could enhance the biological functions of the bacterial community [27]. Until now, the relationship between the metabolic activity of the sediment bacterial community and its composition remains unclear. Meanwhile, the metabolic activity and diversity of the bacterial community are commonly used to evaluate the impact of contaminants on environmental health [28]. It is essential and meaningful to realize the ecotoxicity of MCs during cyanobacteria blooms, based on the variances of metabolic activity and structural changes of the sediment microbial community. Moreover, understanding the dynamic changes in the bacterial community with the existence of MCs may provide crucial information that will be helpful in recognizing and isolating MC-degrading bacteria.

The present study aims to assess the effects of MC-LR in an environmentally-relevant concentration on the properties of sediment bacterial community under anaerobic conditions, including degradation capacity of MC-LR, metabolic profile, and structural diversity. Biolog EcoPlates were used to determine the ecological impact of MC-LR on the sediment bacterial community. The possible connections between the degradation ability of MC-LR and bacterial community composition were revealed, which was conducive to discover more potential bacteria that can decompose MCs under anaerobic conditions.

## 2. Results

### 2.1. Effects on MC-Degrading Ability of Bacterial Community

**Figure 1:**
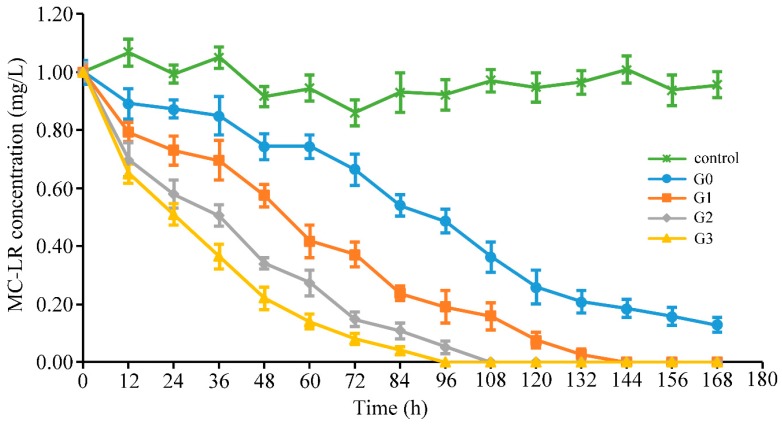
Degradation curves of microcystin-LR (MC-LR) by different samples with different incubation time. Autoclaved sediment bacterial community was used as control to monitor abiotic degradation or adsorption of MC-LR. Data are shown as mean values ± standard deviation (SD) (*n* = 3).

The original bacterial community (sample G0) in the sediment was repeatedly treated with 1 mg/L MC-LR under anaerobic conditions. Sample G1, sample G2, and sample G3, were collected from the experimental system, after 1 week, 2 weeks, and 3 weeks of incubation, respectively. The ability to degrade MC-LR by the continuously treated bacterial community was assessed by measuring the toxin residues in the culture system (Figure 1). In all treatments, the degradation increased as incubation time increased. Sample G3 had the maximum average degradation rate of 0.33 mg/L/day, followed by sample G2, sample G1, and sample G0 of 0.22, 0.18, and 0.12 mg/L/day. The degradation rates of MC-LR increased with toxin addition, as indicated by the decrease in the time required for complete degradation of MC-LR. Successive addition of MC-LR accelerated the degradation ability to nearly three-fold than that of the natural bacterial community during three weeks of incubation. As shown in Figure 1, MC-LR was degraded immediately by all samples without any lag phase, once the toxin was added into experimental system. In the entire degradation process, the degradation rates of MCs were decreased with the decline of the toxin concentration remained in culture system. It was observed that the control group without the addition of the bacterial community did not degrade at all times (Specific values of MC-LR concentration can be seen in Table S1).

### 2.2. Effects on Metabolic Functions of Bacterial Community

**Figure 2:**
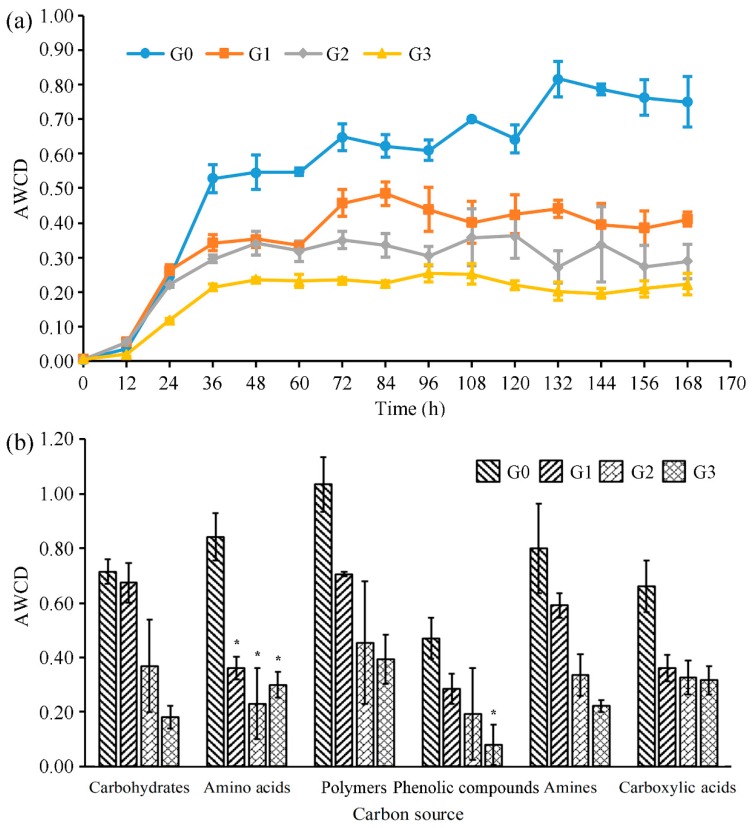
Average well color development (AWCD) changes of sediment bacterial communities with different incubation time (**a**). Utilization of various carbon sources by the sediment bacterial communities in different samples after 168 h of incubation (**b**). Sample G0, which represented the original sediment bacterial community, was used as the control. Data are shown as mean values ± SD (*n* = 3), * *p* < 0.05 (sample G0 versus MC-LR treated samples).

The average well color development (AWCD) value is commonly recommended as an indicator to provide valuable information about the microbial population changes over time [29,30]. The dynamic profiles of the AWCD values in the sediment bacterial communities after repeated treatments of MC-LR are shown in Figure 2a. The AWCD values of the four samples gradually increased, and then reached a plateau with the extension of incubation time in an anaerobic environment. However, the time required for bacterial communities to reach a plateau was shortened. The G3 sample had the minimum time of 36 h, which was only half of the G0 sample. As shown in Figure 2a, the AWCD profiles of all treated samples were lower than those of the control sample G0. Interestingly, this effect was positively correlated with MC-LR treatment times. The overall metabolic activities of bacterial communities decreased by 45.33% after 7 days of MC-LR incubation from sample G0 to G1 in an anaerobic environment. 29.83% and 23.26% of decline in overall metabolic activity was observed from the sample G1 to the sample G2, and from the sample G2 to the sample G3, respectively. These data indicated that MC-LR inhibited the metabolic level of the microbial community in an anaerobic environment, and the reduction of inhibition rates indicated the gradual adaptation of the bacterial community to the external environment.

The Biolog EcoPlate contains 31 carbon sources, which can be divided into 6 types (carbohydrates, amino acids, polymers, phenolic compounds, amines, and carboxylic acids). The metabolic patterns of the bacterial community can be obtained by different utilization capabilities of various carbon sources (Figure 2b). The metabolic capacity of the bacterial communities for 6 types of carbon sources decreased with increasing treatment times, except for the increased amino acid metabolism of sample G3 compared to sample G2, indicating the recovery of amino acid metabolism by the enriched bacterial communities under anaerobic conditions. The utilization proportions of carboxylic acids, polymers, and amino acids in all carbon sources increased from 14.61% to 21.21%, 22.88% to 26.42%, and 18.62% to 20.10%, respectively, compared sample G0 with sample G3. The AWCD values of phenolic compounds, carbohydrates, and amines decreased by 83.17%, 74.83%, and 72.28%, respectively, from the original sample G0 to sample G3, indicating that the metabolic capacity of the bacterial communities for these 3 types of carbon sources was severely inhibited by MC-LR in an anaerobic environment (Specific AWCD values can be seen at Table S2).

### 2.3. Effects on the Diversity and Composition of Bacterial Community

#### 2.3.1. Diversity of the Bacterial Community

Alpha diversity metrics were examined to investigate the intra-group diversity of samples with different culture time at the 97% operational taxonomic units (OTU) level. OTU number, Shannon (a popular diversity index in the ecology proposed by Claude Shannon), Simpson (the diversity index in the ecology introduced by Edward Simpson), Pielou (an indicator to measure species evenness), chao1 (an indicator to measure species richness), abundance-based coverage estimator (ACE), and goods_coverage (an indicator of sequencing depth) indices are listed in Table 1. The original sample G0 had the highest microbial diversity. Sample G1 had the lowest Shannon, Simpson, and Pielou indices. However, sample G2 had the lowest diversity indices of OTU number, Chao1, and ACE. These results indicated that microbial diversity decreased first and then gradually increased as the treatment was prolonged throughout the treatment process. The index of goods_coverage showed that the recovered community effectively represented those in the samples.

**Figure 3:**
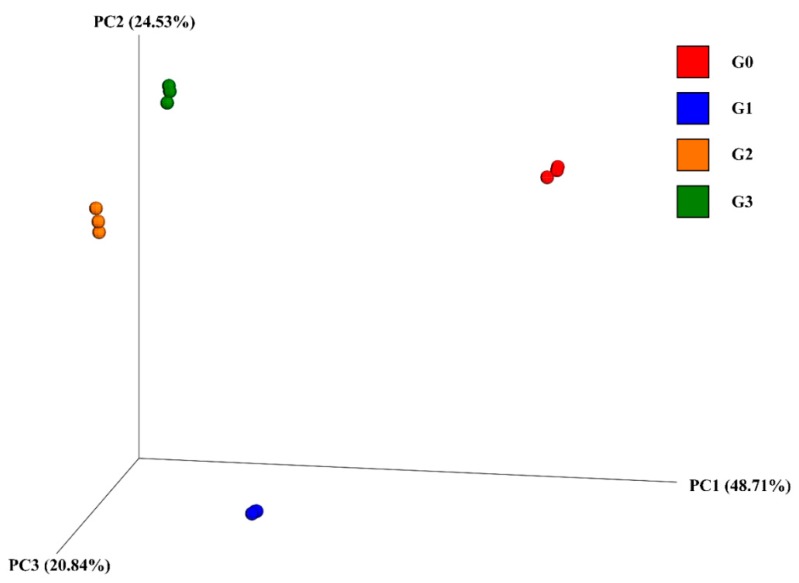
The beta diversity of bacterial communities in different samples. Each point in this figure represents a treated sample and different colors indicate different treated groups (*n* = 3). Beta diversity was visualized using PCoA plot with Bray–Curtis dissimilarity distances.

Beta diversity metrics were used to represent inter-group diversity, and principal coordinate analysis (PCoA) was adopted in this study (Figure 3). The samples were significantly clustered into four groups (G0, G1, G2, and G3), which indicated that MC-LR and anaerobic conditions had a significant effect on the diversity of the bacterial community. Groups G0 and G1 were distantly separated, and groups G2 and G3 were relatively close. Principal coordinate 1 (PC1) explained 48.71% of the variance, PC2 represented 24.53%, and PC3 20.84%. These results suggest that the effect of MC-LR on the sediment bacterial community structure was weakened with the increase in treatment times under anaerobic conditions.

#### 2.3.2. Changes in Bacterial Community Structure

**Figure 4:**
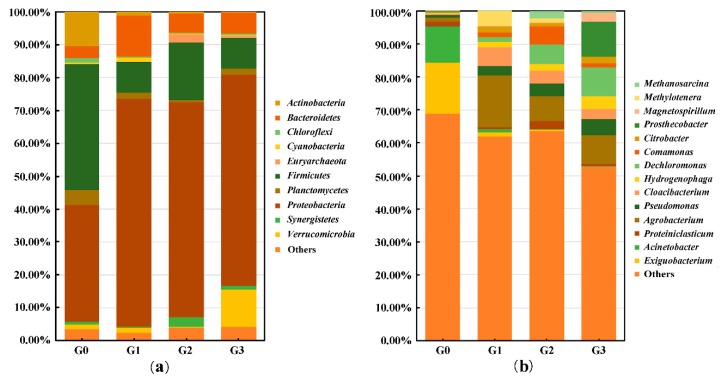
Relative abundances of different groups at the phylum (**a**) and genus (**b**) levels. Phyla (a) and genera (b), whose relative abundance was below 1.00% in all groups or which cannot be assigned to a specific taxonomic category, were grouped into others. The data showed the integrated results of three replicated samples.

The composition of the bacterial community was obtained through 16s rRNA high-throughput sequencing. A total of 43 phyla were annotated among all samples, and 10 phyla whose relative abundance was higher than 1.00% in at least one group were selected for comparison (Figure 4). As shown in Figure 4a, in group G0, *Firmicutes* (38.11%), *Proteobacteria* (35.44%), and *Actinobacteria* (10.51%) were the main phyla, accounting for 84.06% of the total relative abundance. In group G3, the dominant phyla were *Proteobacteria* (64.16%), *Verrucomicrobia* (11.36%), and *Firmicutes* (9.41%), which accounted for 84.94% of the total bacteria. The relative abundance of *Proteobacteria* increased nearly twofold during the first incubation from group G0 to group G1, and then remained consistently high. However, the relative abundance of *Firmicutes* maintained a general declining trend, and that of *Actinobacteria* decreased continuously during the entire process. It should be noted that the abundance of *Verrucomicrobia* rapidly increased from group G2 to group G3.

**Figure 5:**
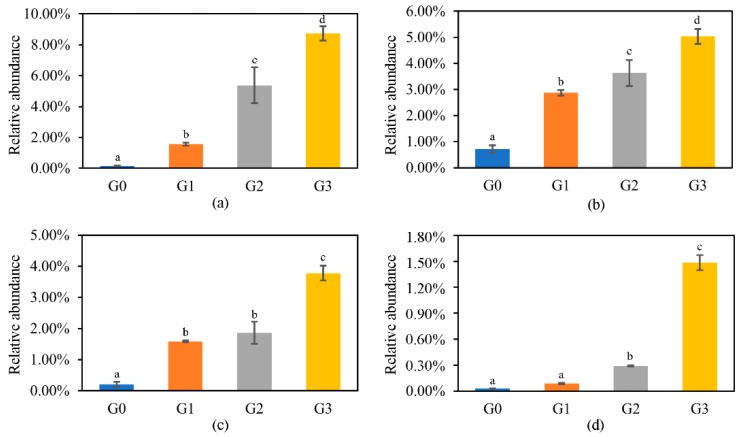
The changes in relative abundance in genera whose proportion continuously increased with increased treatment times: (**a**) *Dechloromonas*, (**b**) *Pseudomonas*, (**c**) *Hydrogenophaga*, and (**d**) *Magnetospirillum.* Data are shown as mean values ± SD (*n* = 3) and letters up the bars show significant statistical difference according to LSD test (*p* < 0.05).

At the genus level, 14 genera whose abundance was greater than 1.00% in at least one group were chosen for inert-group comparison (Figure 4b). The dominant genera in group G0 were *Exiguobacterium* (15.51%), and *Acinetobacter* (10.84%). However, in group G3, *Prosthecobacter* (10.52%), *Dechloromonas* (8.72%), and *Agrobacterium* (8.62%) had the highest relative abundance, accounting for 27.86% of the total genera. Figure 5 shows the genera whose relative abundance increased with the prolongation of treatment times, and the overall increment was greater than 1.00%. *Dechloromonas* had the maximum increasing multiples (54.63), followed by *Magnetospirillum* (50.10), *Hydrogenophaga* (18.62), and *Pseudomonas* (7.03).

**Figure 6:**
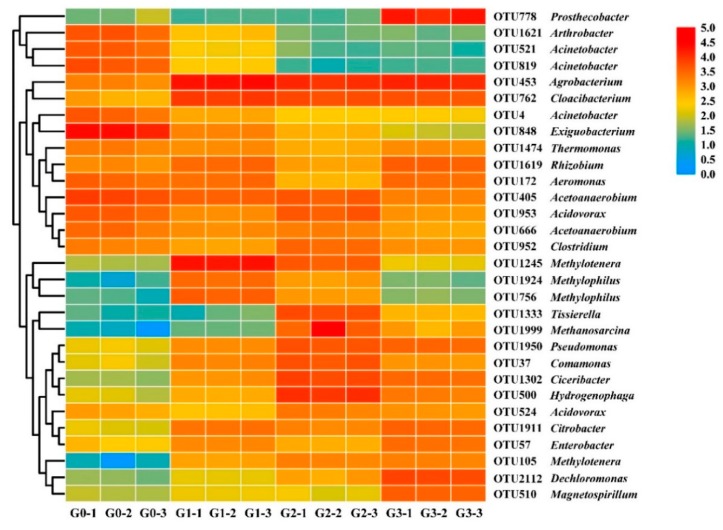
Heatmap of the 30 most abundant operational taxonomic units (OTUs) at the genus level in all samples. The OTUs were annotated with taxonomic names based on the BLAST of the extracted genetic sequences.

The 30 greatest operational taxonomic units (OTUs) at the genus level were selected for heatmap. There were four types of variation trend pertaining to the relative abundance of bacteria, which can be observed from the cluster analysis (Figure 6); (1) the relative abundance of *Arthrobacter* (OTU1621), *Acinetobacter* (OTU521, OTU819, and OTU4), and *Exiguobacterium* (OTU848) decreased; (2) the relative abundances of *Tissierella* (OTU1333), *Methanosarcina* (OTU1999), *Pseudomonas* (OTU1950), *Comamonas* (OTU37), *Ciceribacter* (OTU1302), *Hydrogenophaga* (OTU500), *Acidovorax* (OTU524), *Citrobacter* (OTU1911), *Enterobacter* (OTU57), *Methylotenera* (OTU105), *Dechloromonas* (OTU2112), and *Magnetospirillum* (OTU510) increased overall; (3) from OTU1474 to OTU952, the relative abundance remained comparably high in all samples; (4) the relative abundance of *Methylotenera* (OTU1245) and *Methylophilus* (OTU1924 and OTU756) increased from group G0 to group G1, then gradually declined. In addition, *Agrobacterium* and *Cloacibacterium* were maintained at high levels of relative abundance after the rapid increase from group G0 to group G1. *Prosthecobacter* maintained an extremely low percentage from the original bacterial community to group G2, while a sudden increase occurred during the incubation from group G2 to group G3, which explained the changes in *Verrucomicrobia* at the corresponding time. 

## 3. Discussion

The aim of this study was to uncover the effect of MCs on the structure and metabolic function of the sediment bacterial community under anaerobic conditions, which is widespread in the natural environment. Enhanced biodegradation of MC-LR was verified by the decrease in time required for complete degradation, and the degradation capacity was positively correlated with the MC-LR treatment frequency. Comparing groups G1 to G0, the degradation rate of MC-LR was significantly increased, which may be caused by the proliferation of MC-degrading microbial populations and the inhibition of sensitive bacteria. These sensitive microbial populations cannot utilize MCs as sole carbon and nitrogen sources, and the selective pressure of MC-LR induced them to decrease rapidly. Comparing the second and third treatments to the first treatment, the rate of increase in the degradation capacity declined, which may be attributable to the gradual stabilization of the bacterial community. Therefore, repeated exposure of MCs enhanced the biodegradation capacity of the bacterial community through enriching functional bacteria. There was no lag phase observed in the degradation processes of all groups, which reflected the extensive existence of MC-degrading bacteria in the original microbial community [31]. Wu et al. found that dissolved MCs could be degraded by lake sediments after 2–6 days of lag phase under anoxic conditions [13]. The degradation capacity of a specific chemical by the microbial community can be considered as a relevant indicator of exposure to this pollutant in the natural environment [32]. This was considerable in Lake Taihu, which was plagued by cyanobacteria and cyanotoxins for many years [8,33]. Therefore, the long selective pressure of MCs changed the structure of the sediment microbial community towards greater efficiency in the biodegradation of MCs. So far, most of the MC-degrading bacteria are aerobic microbes and few anaerobic functional bacteria were reported [10]. The degradation capability of these aerobic functional bacteria was varied from 1.5 µg/L/day to 60 mg/L/day and the optimal degrading condition are 20–30 °C, neutral or slight alkaline [31]. Huang et al. isolated one anaerobic MC-degrading bacteria, *Enterobacter* sp. YF3 with the maximum degradation rate of 0.34 mg/L/day under the optimal condition (37 °C, pH = 7, 5 mg/L MC-LR) [34]. Bao and Wu found that amino acid-degrading anaerobic bacterium ALA-1 had greater degrading ability under mesophilic and alkaline conditions than cold, neutral, and slightly acidic conditions [35]. Zhu et al. deduced that *Candidatus Cloacamonas acidaminovorans* Evry may degrade MC-LR under anaerobic conditions through microbial community analysis [36].

The effect of MCs on the metabolic functions of the bacterial community was assessed using the Biolog EcoPlate, which was effective at distinguishing the changes in the metabolism of microbial communities [37,38]. The total metabolic capacity and utilization of six types of carbon sources were inhibited, which may be caused by the inhibitory effect of MC-LR on the bacterial population, accounting for the metabolism of these carbon sources. Similar to this study, Cao et al. found that high concentrations of MC-LR inhibited the overall carbon metabolic activity of the bacterial community and formed different patterns of carbon utilization in typical lakeside soils [28]. The extent of reduction in metabolic capacity decreased with prolonged treatment times, with the same variation trends in degradation rate. The gradual stabilization of bacterial community structure may explain this phenomenon. Interestingly, the metabolic capacity of amino acids recovered in group G3, and this may be due to the chemical property of MC-LR, which belongs to a special type of amino acid [31,39]. 

The alpha diversity decreased to the lowest values at the early stage and then recovered from group G1 or G2. The first decline may be caused by the decrease in the bacterial population, which cannot utilize MCs as nutritious sources. Therefore, their abundance decreased to minimum during 1 to 2 weeks of coculture with MCs. Bacteria that were able to degrade or resist MCs then rapidly proliferated using MCs and/or their metabolites as growth substances. Su et al. found that the alpha diversity of bacterial communities in the water column was reduced by *Microcystis* abundance and MC concentrations, which was consistent with the current study results at the early stage [18]. A similar suppression–recovery pattern of the diversity of bacterial communities was also observed in the microcosm treated by other chemicals [40]. PCoA revealed a significant effect of MC treatment on the inter-group diversity of the bacterial community. This could be due to the combined effect of the changes in abundances of MCs-sensitive bacteria and MCs-degrading bacteria.

At the phylum level, the abundance of *Proteobacteria* rapidly increased and remained the most abundant phylum, which indicated that most MC-degrading bacteria might be present at this phylum. However, *Firmicutes* and *Actinobacteria*, the main categories in the environmental samples, decreased significantly with the increased treatments. These two phyla could either be sensitive to MC-LR or could not achieve sufficient nutrients to maintain their metabolism and survival. Ma et al. also detected that *Proteobacteria* was the most dominant species during the anoxic biodegradation of MC-LR in drinking-water sludge [41,42]. 

At the genus level, the variation trends of the 30 most abundant OTUs were versatile. Bacteria whose abundance obviously increased with increasing treatment frequency, might be able to metabolize MCs or their metabolic products as carbon and nitrogen sources. Dozens of MC-degrading bacteria have been isolated and identified from nature in aerobic environments, but few functional bacteria under anaerobic conditions [3,10,34]. The repeated treatment of MCs to sediment bacterial community in the anaerobic environment can be regarded as an enrichment process of anaerobic MC-degrading bacteria. These enriched bacterial populations may be an important source of potential MC-degrading bacteria in anaerobic environments. *Dechloromonas* had the maximum proliferation of relative abundance during the entire treatment process. Chakraborty and Salinero reported the metabolic versatility of *Dechloromonas*, including degradation of benzene, toluene, ethylbenzene, xylene, and perchlorate, anaerobically through hydroxylation and carboxylation [43,44,45]. Therefore, *Dechloromonas* is an important biological resource to degrade refractory pollutants and more attention should be paid to field application. It was reported that *Pseudomonas* could degrade MC-LR at a rate of 0.055 to 2500 μg/L/day and lyse *Microcystis aeruginosa* under aerobic conditions [46,47,48]. The abundance of *Pseudomonas* continued to increase with continuous MC-LR treatment. Similar to our results, Ma et al. maintained that *Pseudomonas* could also degrade MCs under anoxic conditions through the correlation analysis of bacterial abundance and degraded number of MCs [41]. *Hydrogenophaga* prefers carboxylic acids and amino acids as nutrient sources [49]. The increase in anaerobic fermentation products of microorganisms and metabolites of MCs may induce an increase in *Hydrogenophaga*. *Magnetospirillum* is usually located at the sediment-water interface and is reported as a denitrifying toluene degrader under anaerobic conditions [50]. *Magnetospirillum* may utilize MCs and/or their metabolites as new types of growth substances. *Agrobacterium*, is capable of degrading varieties of chemical substances, including organophosphorus herbicides and pesticides (atrazine, methyl parathion, etc.), 3-hydroxypyridine, p-nitrophenol, and nicotine; and may be a potential MC-degrading bacterium [51,52,53,54].

MC-degrading bacteria exist in the natural environment extensively, such as *Sphingomonas* sp. ACM-3962, *Sphingopyxis* sp. m6, *Enterobacter* sp. YF3, and etc. [10,39]. Due to the combined effect of these functional bacteria, the MC concentration maintains at a relatively low level in the natural environment. However, Henao et al. found MCs could be detected in sediment cores through paleolimnological investigations, indicating that these MC-degrading bacteria are unable to completely degrade all of toxins under environmental conditions [14]. The variability of natural climate may affect the degradation of MCs by functional bacteria in the natural environment. In addition, it was reported that MCs can be produced locally by benthic cyanobacteria and buried *Microcystis* colonies at the lake bottom [55]. 

The *mlr* gene cluster is acknowledged as MC-degrading gene, comprising *mlrA*, *mlrB*, *mlrC*, and *mlrD*, which can express *Mlr* enzymes to sequentially degrade MC-LR, linearized MC-LR, and tetrapeptide [56,57]. Among them, *mlrA* is regarded as the most important one, which encode metalloprotease *MlrA* [58]. Bourne et al. first found that microcystinase *MlrA* can hydrolyze cyclic MC-LR to linearized MC-LR through breaking the peptide bond at Arg-Adda [56,57]. Due to *mlrA* gene exist in most aerobic MC-degrading bacteria, it is regarded as the criterion of MC-degrading bacteria. Therefore, *mlrA* gene should be further amplified to identify whether it is contained in the anaerobic MC-degrading bacteria. Chen et al. found that MC-LR could be degraded by lake sediments under anoxic conditions with the production of final product Adda [25]. Linearized MC-LR, tetrapeptide, and adda, known as aerobic degradation products of MC-LR, were also produced by bacterium ALA-1 [35]. Two new linearized MC-LR, Ala-Leu-MeAsp-Arg-Adda-Glu-Mdha and Leu-MeAsp-Arg-Adda-Glu-Mdha-Ala, were detected in the degradation products of MC-LR, proving that new degradation pathways exit in anaerobic MC-degrading bacteria through hydrolyzing peptide bonds at Ala-Mdha and Ala-Leu [36,59]. In this study, linearized MC-LR, tetrapeptide, and adda were also detected (data was not shown) by LC/MS/MS. However, the chemical structure of these products should be further studied to illustrate specific degradation pathway of MCs under anaerobic conditions. 

## 4. Conclusions

This is the first study to explore the effect of MCs on the metabolic function and structure succession of the bacterial community from eutrophic lake sediment under anaerobic conditions. In this study, the sediment bacterial community was repeatedly treated by MC-LR, and metabolic function and community composition were analyzed throughout the entire process. The data indicated that the MC-degrading capacity of the bacterial community was clearly improved with increased treatment times. However, a biological assay showed that overall carbon metabolic activity gradually decreased. A trend of suppression–recovery on microbial diversity indices was also found with increased treatment frequency. Furthermore, the structure of the bacterial community was significantly changed owing to repeated treatment with MC-LR. Four potential MC-degrading bacterial genera under anaerobic conditions were found: *Dechloromonas*, *Pseudomonas*, *Hydrogenophaga*, and *Magnetospirillum*. 

## 5. Materials and Methods

### 5.1. Sediment Samples and Reagents

Three different sediment samples (under surface 10–15 cm) were collected from Fudu Bay of Lake Taihu, China, where heavy cyanobacterial blooms occur frequently [33,60]. These samples were packaged in sterile anaerobic bags and transported to the laboratory rapidly at low temperatures to guarantee the activity of the bacterial community. 

MC-LR standards with purity ≥ 95% were purchased from Enzo Life Sciences Inc (Farmingdale, NY, USA). Acetonitrile (Merck, Darmstadt, Germany) and formic acid (Fisher Scientific, Shanghai, China) of mass spectrometric grade were used to detect MC-LR. Phosphate buffer solution (PBS, Beyotime, Shanghai, China) was used to wash the bacterial body and dilution. 

### 5.2. Obtain of Bacterial Community and Experimental Setup

Solid debris was removed manually, and three sediment samples were pooled together evenly. Thirty grams of mixed sediment was added to a 500 mL flask containing 270 mL of autoclaved lake water, collected from the same sampling sites. The sediment sample in the flask was detached through constant shaking at 25 °C and 120 rpm for 30 min. After 10 min of standing, 100 mL of supernatant was transferred and centrifuged to collect bacteria (5000× *g*, 4 °C, 10 min). The collected bacteria were washed twice using sterile PBS to remove the interference of other substances, and then inoculated into a new 250 mL flask containing 100 mL sterile water as the original bacterial community of sediment (sample G0). All of these procedures were completed in an anaerobic incubator.

Based on the previous results about MCs concentration in sediment, the maximum concentration was up to 0.78 mg/kg in Lake Yangebup, 0.73 mg/kg in Lake Baptiste, and 0.83 mg/kg in Lake Lac Waterloo [16,21]. Therefore, a final concentration of 1 mg/L standard MC-LR was added into the experimental system (sample G0) to simulate the maximum environmental exposure of MCs in lake sediment. This culture system was incubated in a dark shaker at 25 °C and 120 rpm under anaerobic conditions. After cultivation for one week, a new bacterial community was generated as sample G1. Standard MC-LR was then added into sample G1 to a final concentration of 1 mg/L and cultivated for another week. Samples G2 and G3 were generated following the same method to analyze the interaction between MC-LR and the bacterial community in different phases. Autoclaved sample G0 was used as the control for non-biological degradation or adsorption of MC-LR. Sample G0 was used as the control to detect the original metabolic function and bacterial community structure. All the samples had three replicates and the experiments were carried out in triplicate. The anaerobic condition was obtained through the adsorption of oxygen by an anaerobic pack (Oxide, Hants, UK). The oxygen in the sealed anaerobic jar (Oxide, Hants, UK) could be adsorbed by the anaerobic pack in a few minutes. The concentration of oxygen was verified by an anaerobic indicator (Thermo scientific, Hants, UK).

### 5.3. Detection of MC-LR

One hundred microliters of well-mixed culture was collected every 24 h to detect the concentration of MC-LR. Collected samples were centrifuged at 12,000× *g* for 15 min at 4 °C. 80 μL of the supernatant was then transferred into sample vials for LC-MS/MS detection. The specific methods and instrumental parameters were identical to those previously described [31].

### 5.4. Metabolic Ability of Bacterial Community

In an anaerobic incubator, 1 mL of the collected sample was transferred to a tube containing 10 mL PBS and serially diluted to form a 10^−3^ dilution. The diluted solution was well-blended, and 150 μL of them were inoculated into every micropore of the Biolog EcoPlate (Biolog Inc. Hayward, CA, USA) using an 8-channel pipette. The plates were then incubated at 25 °C for 7 days under anaerobic conditions. The absorbance values were measured rapidly at 590 nm and 750 nm using a microplate reader at 0, 24, 48, 72, 96, 120, 144, and 168 h. The AWCD and metabolic ability of different carbon sources were calculated according to the methods described by Cao et al. [28]. The AWCD value indicates the metabolic activity of the overall microbial community. Each micropore has three replicates and 31 of the most common carbon sources were used to evaluate the functional diversity of community-level heterotrophic bacterial assemblages. 

### 5.5. DNA Extraction and Sequencing

DNA of samples G0, G1, G2, and G3, collected at 0, 7, 14, and 21 days, were extracted according to a previously reported method [39]. The V3–V4 region of 16S rDNA was then amplified using the specific primers with barcodes 341F (5′-CCTACGGGNGGCWGCAG-3′) and 806R (5′-GGACTACHVGGGTATCTAAT-3′). The amplification products were separated by electrophoresis on a 2% agarose gel, and the target amplicons were collected using a DNA gel extraction kit (Axygen, CA, USA). These purified amplicons were mixed equivalently to construct a library for further sequencing by Illumina Hiseq 2500.

### 5.6. Analysis of the Bacterial Community Structure

Filtering, splicing, chimera-removing, and other treatments of raw sequencing data were performed according to a previously reported protocol [61,62]. Generally, 1,944,980 valid sequences were obtained with an average of 162,081 ± 9542 sequence reads. The filtered clean tags were clustered and classified into OTUs using USEARCH software (version 11.0.667, Edgar, R.C., Tiburon, CA, USA), with the UCLUST algorithm at 97% sequence similarity. The alpha diversity indices (Shannon, Simpson, Pielou, Chao1, ACE and goods_coverage) were conducted using R software (version 3.5.3, Lucent Technologies, Inc., Murray Hill, NJ, USA) with the vegan package. The beta diversity analysis (PCoA) was performed by QIIME2 (version 2019.10, University of Colorado, Boulder, CO, USA) to visualize the differences in bacterial communities treated by MC-LR. A total of 2132 OTUs were annotated with taxonomic classifications, based on the Greengene database (version gg_13_5, Second Genome, Inc., San Francisco, CA, USA). Specific characteristics of species abundance at different levels were also conducted using QIIME2 software.

### 5.7. Statistical Analysis

Means ± SD were adopted to interpret the results in this study. One-way analysis of variance (ANOVA) was applied to distinguish differences among samples if the values were normally distributed and variances were homogenous. In case the values were not normally distributed or variances were heterogenous, the nonparametric Mann−Whitney *U* test was applied using SPSS (version 15.0, IBM, Armonk, NY, USA) and *p* < 0.05 was considered significant.

## Figures and Tables

**Table 1:** Alpha diversity indices of the bacterial communities in different samples.

Samples	OTU Number	Shannon	Simpson	Pielou	Chao1	ACE	Goods_Coverage
G0	1369 ± 31.19^a^	7.22 ± 0.36^a^	0.97 ± 0.01	0.69 ± 0.04^a^	1498.62 ± 16.88^a^	1470.41 ± 31.44^a^	99.91% ± 0.00%^a^
G1	1045 ± 11.36^b^	6.34 ± 0.06^b^	0.95 ± 0.00	0.63 ± 0.00^b^	1235.11 ± 59.95^b^	1205.59 ± 26.19^b^	99.87% ± 0.02%^b^
G2	844 ± 33.78^c^	6.38 ± 0.35^b^	0.96 ± 0.02	0.66 ± 0.03	1089.2 ± 85.93^c^	1043.07 ± 58.28^c^	99.89% ± 0.02%
G3	1025.33 ± 25.01^b^	6.72 ± 0.08^b^	0.97 ± 0.00	0.67 ± 0.01	1223.26 ± 36.18^b^	1186.05 ± 41.49^b^	99.88% ± 0.00%

Data were shown as mean values ± SD (*n* = 3) and different letters followed represented significantly statistical difference (*p* < 0.05).

## Supplementary Materials

The following are available online at https://www.mdpi.com/2072-6651/12/3/183/s1, Table S1: The concentrations of MC-LR for different samples with different incubation time; Table S2: Utilization of various carbon sources by the sediment bacterial communities in different samples after 168 h of incubation.

## References

[1] Huisman J., Codd G.A., Paerl H.W., Ibelings B.W., Verspagen J.M.H., Visser P.M. (2018). Cyanobacterial blooms. Nat. Rev. Microbiol..

[2] Paerl H.W., Otten T.G. (2013). Blooms bite the hand that feeds them. Science.

[3] Massey I.Y., Zhang X., Yang F. (2018). Importance of bacterial biodegradation and detoxification processes of microcystins for environmental health. J. Toxicol. Environ. Health B.

[4] Zilliges Y., Kehr J.C., Meissner S., Ishida K., Mikkat S., Hagemann M., Kaplan A., Borner T., Dittmann E. (2011). The cyanobacterial hepatotoxin microcystin binds to proteins and increases the fitness of *Microcystis* under oxidative stress conditions. PLoS ONE.

[5] Dittmann E., Erhard M., Kaebernick M., Scheler C., Neilan B.A., von Dohren H., Borner T. (2001). Altered expression of two light-dependent genes in a microcystin-lacking mutant of *Microcystis aeruginosa* PCC 7806. Microbiology.

[6] Schatz D., Keren Y., Vardi A., Sukenik A., Carmeli S., Borner T., Dittmann E., Kaplan A. (2007). Towards clarification of the biological role of microcystins, a family of cyanobacterial toxins. Environ. Microbiol..

[7] Hu C., Rzymski P. (2019). Programmed cell death-like and accompanying release of microcystin in freshwater bloom-forming cyanobacterium *Microcystis*: From identification to ecological relevance. Toxins.

[8] Yang F., Massey I.Y., Guo J., Yang S., Pu Y.P., Zeng W.M., Tan H.Z. (2018). Microcystin-LR degradation utilizing a novel effective indigenous bacterial community YFMCD1 from Lake Taihu. J. Toxicol. Environ. Health A.

[9] Chen L., Xie P. (2016). Mechanisms of microcystin-induced cytotoxicity and apoptosis. Mini-Rev. Med. Chem..

[10] Li J.M., Li R.H., Li J. (2017). Current research scenario for microcystins biodegradation—A review on fundamental knowledge, application prospects and challenges. Sci. Total Environ..

[11] Shimizu K., Sano T., Kubota R., Kobayashi N., Tahara M., Obama T., Sugimoto N., Nishimura T., Ikarashi Y. (2014). Effects of the amino acid constituents of microcystin variants on cytotoxicity to primary cultured rat hepatocytes. Toxins.

[12] Chen L., Giesy J.P., Xie P. (2018). The dose makes the poison. Sci. Total Environ..

[13] Wu X.Q., Wang C.B., Tian C.C., Xiao B.D., Song L.R. (2015). Evaluation of the potential of anoxic biodegradation of intracellular and dissolved microcystins in lake sediments. J. Hazard. Mater..

[14] Henao E., Rzymski P., Waters M.N. (2019). A review on the study of cyanotoxins in paleolimnological research: Current knowledge and future needs. Toxins.

[15] Brunberg A.K., Blomqvist P. (2002). Benthic overwintering of *Microcystis* colonies under different environmental conditions. J. Plankton Res..

[16] Song H., Coggins L.X., Reichwaldt E.S., Ghadouani A. (2015). The importance of lake sediments as a pathway for microcystin dynamics in shallow eutrophic lakes. Toxins.

[17] Lezcano M.A., Velazquez D., Quesada A., El-Shehawy R. (2017). Diversity and temporal shifts of the bacterial community associated with a toxic cyanobacterial bloom: An interplay between microcystin producers and degraders. Water Res..

[18] Su X.M., Steinman A.D., Tang X.M., Xue Q.J., Zhao Y.Y., Xie L.Q. (2017). Response of bacterial communities to cyanobacterial harmful algal blooms in Lake Taihu, China. Harmful Algae.

[19] Parulekar N.N., Kolekar P., Jenkins A., Kleiven S., Utkilen H., Johansen A., Sawant S., Kulkarni-Kale U., Kale M., Saebo M. (2017). Characterization of bacterial community associated with phytoplankton bloom in a eutrophic lake in South Norway using 16S rRNA gene amplicon sequence analysis. PLoS ONE.

[20] Chen W., Song L.R., Peng L., Wan N., Zhang X.M., Gan N.Q. (2008). Reduction in microcystin concentrations in large and shallow lakes: Water and sediment-interface contributions. Water Res..

[21] Zastepa A., Pick F.R., Blais J.M., Saleem A. (2015). Analysis of intracellular and extracellular microcystin variants in sediments and pore waters by accelerated solvent extraction and high performance liquid chromatography-tandem mass spectrometry. Anal. Chim. Acta.

[22] Maerki M., Muller B., Dinkel C., Wehrli B. (2009). Mineralization pathways in lake sediments with different oxygen and organic carbon supply. Limnol. Oceanogr..

[23] Yuan X.Z., Shi X.S., Zhang D.L., Qiu Y.L., Guo R.B., Wang L.S. (2011). Biogas production and microcystin biodegradation in anaerobic digestion of blue algae. Energ. Environ. Sci..

[24] Conrad R., Chan O.C., Claus P., Casper P. (2007). Characterization of methanogenic Archaea and stable isotope fractionation during methane production in the profundal sediment of an oligotrophic lake (Lake Stechlin, Germany). Limnol. Oceanogr..

[25] Chen X.G., Yang X., Yang L.L., Xiao B.D., Wu X.Q., Wang J.T., Wan H.G. (2010). An effective pathway for the removal of microcystin LR via anoxic biodegradation in lake sediments. Water Res..

[26] Holst T., Jorgensen N.O.G., Jorgensen C., Johansen A. (2003). Degradation of microcystin in sediments at oxic and anoxic, denitrifying conditions. Water Res..

[27] Wu Y.C., Cai P., Jing X.X., Niu X.K., Ji D.D., Ashry N.M., Gao C.H., Huang Q.Y. (2019). Soil biofilm formation enhances microbial community diversity and metabolic activity. Environ. Int..

[28] Cao Q., Steinman A.D., Su X., Xie L. (2017). Effects of microcystins contamination on soil enzyme activities and microbial community in two typical lakeside soils. Environ. Pollut..

[29] Garland J.L. (1997). Analysis and interpretation of community-level physiological profiles in microbial ecology. FEMS Microbiol. Ecol..

[30] Preston-Mafham J., Boddy L., Randerson P.F. (2002). Analysis of microbial community functional diversity using sole-carbon-source utilisation profiles—A critique. FEMS Microbiol. Ecol..

[31] Ding Q., Liu K.Y., Xu K., Sun R.L., Zhang J., Yin L.H., Pu Y.P. (2018). Further understanding of degradation pathways of microcystin-LR by an indigenous *Sphingopyxis* sp. in environmentally relevant pollution concentrations. Toxins.

[32] Gallego S., Devers-Lamrani M., Rousidou K., Karpouzas D.G., Martin-Laurent F. (2019). Assessment of the effects of oxamyl on the bacterial community of an agricultural soil exhibiting enhanced biodegradation. Sci. Total Environ..

[33] Yang F., Zhou Y.L., Sun R.L., Wei H.Y., Li Y.H., Yin L.H., Pu Y.P. (2014). Biodegradation of microcystin-LR and-RR by a novel microcystin-degrading bacterium isolated from Lake Taihu. Biodegradation.

[34] Huang F., Feng H., Li X., Yi X., Guo J., Clara T., Yang F. (2019). Anaerobic degradation of microcystin-LR by an indigenous bacterial *Enterobacter* sp. YF3. J. Toxicol. Environ. Health A.

[35] Bao Z., Wu Y. (2014). Biodegradation of microcystin-LR by an amino acid-degrading anaerobic bacterium. Desalin. Water Treat..

[36] Zhu F.P., Han Z.L., Duan J.L., Shi X.S., Wang T.T., Sheng G.P., Wang S.G., Yuan X.Z. (2019). A novel pathway for the anaerobic biotransformation of microcystin-LR using enrichment cultures. Environ. Pollut..

[37] Guo P.P., Zhu L.S., Wang J.H., Wang J., Liu T. (2015). Effects of alkyl-imidazolium ionic liquid [Omim]Cl on the functional diversity of soil microbial communities. Environ. Sci. Pollut. R.

[38] Lear G., Bellamy J., Case B.S., Lee J.E., Buckley H.L. (2014). Fine-scale spatial patterns in bacterial community composition and function within freshwater ponds. ISME J..

[39] Zhang J., Lu Q.Q., Ding Q., Yin L.H., Pu Y.P. (2017). A novel and native microcystin-degrading bacterium of *Sphingopyxis* sp isolated from Lake Taihu. Int. J. Environ. Res. Public Health.

[40] Fang H., Lian J.J., Wang H.F., Cai L., Yu Y.L. (2015). Exploring bacterial community structure and function associated with atrazine biodegradation in repeatedly treated soils. J. Hazard. Mater..

[41] Ma G.X., Pei H.Y., Hu W.R., Xu X.C., Ma C.X., Li X.Q. (2014). The removal of cyanobacteria and their metabolites through anoxic biodegradation in drinking water sludge. Bioresour. Technol..

[42] Ma G.X., Pei H.Y., Hu W.R., Xu X.C., Ma C.X., Pei R.T. (2016). Effects of glucose on microcystin-LR removal and the bacterial community composition through anoxic biodegradation in drinking water sludge. Environ. Technol..

[43] Chakraborty R., O’Connor S.M., Chan E., Coates J.D. (2005). Anaerobic degradation of benzene, toluene, ethylbenzene, and xylene compounds by *Dechloromonas* strain RCB. Appl. Environ. Microb..

[44] Chakraborty R., Coates J.D. (2005). Hydroxylation and carboxylation-two crucial steps of anaerobic benzene degradation by *Dechloromonas* strain RCB. Appl. Environ. Microb..

[45] Salinero K.K., Keller K., Feil W.S., Feil H., Trong S., Di Bartolo G., Lapidus A. (2009). Metabolic analysis of the soil microbe *Dechloromonas aromatica* str. RCB: Indications of a surprisingly complex life-style and cryptic anaerobic pathways for aromatic degradation. BMC Genom..

[46] Takenaka S., Watanabe M.F. (1997). Microcystin LR degradation by *Pseudomonas aeruginosa* alkaline protease. Chemosphere.

[47] Kang Y.H., Park C.S., Han M.S. (2012). *Pseudomonas aeruginosa* UCBPP-PA14 a useful bacterium capable of lysing *Microcystis aeruginosa* cells and degrading microcystins. J. Appl. Phycol..

[48] Lemes G.A.F., Kist L.W., Bogo M.R., Yunes J.S. (2015). Biodegradation of [D-Leu(1)] microcystin-LR by a bacterium isolated from sediment of Patos Lagoon estuary, Brazil. J. Venom. Anim Toxins.

[49] Magic-Knezev A., Wullings B., Van der Kooij D. (2009). *Polaromonas* and *Hydrogenophaga* species are the predominant bacteria cultured from granular activated carbon filters in water treatment. J. Appl. Microbiol..

[50] Meyer-Cifuentes I., Fiedler S., Muller J.A., Kappelmeyer U., Mausezahl I., Heipieper H.J. (2017). Draft genome sequence of *Magnetospirillum* sp. strain 15-1, a denitrifying toluene degrader isolated from a planted fixed-bed reactor. Microbiol. Resour. Ann..

[51] Zhao S.X., Hu C.H., Guo L.Z., Li K.R., Yu H. (2019). Isolation of a 3-hydroxypyridine degrading bacterium, *Agrobacterium* sp. DW-1, and its proposed degradation pathway. AMB Express.

[52] Wang S.H., Zhang C., Yan Y.C. (2012). Biodegradation of methyl parathion and p-nitrophenol by a newly isolated *Agrobacterium* sp strain Yw12. Biodegradation.

[53] Wang S.N., Liu Z., Xu P. (2009). Biodegradation of nicotine by a newly isolated *Agrobacterium* sp strain S33. J. Appl. Microbiol..

[54] Struthers J.K., Jayachandran K., Moorman T.B. (1998). Biodegradation of atrazine by *Agrobacterium radiobacter* J14a and use of this strain in bioremediation of contaminated soil. Appl. Environ. Microb..

[55] Misson B., Sabarta M., Amblard C., Latour D. (2012). Benthic survival of *Microcystis*: Long-term viability and ability to transcribe microcystin genes. Harmful Algae.

[56] Bourne D.G., Jones G.J., Blakeley R.L., Jones A., Negri A.P., Riddles P. (1996). Enzymatic pathway for the bacterial degradation of the cyanobacterial cyclic peptide toxin microcystin LR. Appl. Environ. Microbiol..

[57] Bourne D.G., Riddles P., Jones G.J., Smith W., Blakeley R.L. (2001). Characterisation of a gene cluster involved in bacterial degradation of the cyanobacterial toxin microcystin LR. Environ. Toxicol.

[58] Dziga D., Tokodi N., Backovic D.D., Kokocinski M., Antosiak A., Puchalski J., Strzalka W., Madej M., Meriluoto J., Svircev Z. (2019). The Effect of a combined hydrogen peroxide-*MlrA* treatment on the phytoplankton community and microcystin concentrations in a mesocosm experiment in Lake Ludos. Toxins.

[59] Ouyang L. (2014). Enzymatic pathway for MCLR degradation by bacterium CJ5. Master’s Thesis.

[60] Tang X., Krausfeldt L.E., Shao K., LeCleir G.R., Stough J.M.A., Gao G., Boyer G.L., Zhang Y., Paerl H.W., Qin B. (2018). Seasonal gene expression and the ecophysiological implications of toxic *Microcystis aeruginosa* blooms in Lake Taihu. Environ. Sci. Technol..

[61] Chiodini R.J., Dowd S.E., Chamberlin W.M., Galandiuk S., Davis B., Glassing A. (2015). Microbial population differentials between mucosal and submucosal intestinal tissues in advanced crohn’s disease of the ileum. PLoS ONE.

[62] Glassing A., Dowd S.E., Galandiuk S., Davis B., Jorden J.R., Chiodini R.J. (2015). Changes in 16s RNA gene microbial community profiling by concentration of prokaryotic DNA. J. Microbiol. Methods.

