# Supplementary material for: Effects of Microcystin-LR on Metabolic Functions and Structure Succession of Sediment Bacterial Community under Anaerobic Conditions
Source: Toxins (Basel). 2020 Mar 15;12(3):183. doi: 10.3390/toxins12030183 (PMC7150748; doi:10.3390/toxins12030183)
Supplement: Supplementary file 1 [file toxins-12-00183-s001.pdf]

Supplementary Materials: Effects of Microcystin-LR on Metabolic Functions and Structure Succession of Sediment Bacterial Community under Anaerobic Conditions

Qin Ding, Kaiyan Liu, Zhiquan Song, Rongli Sun, Juan Zhang, Lihong Yin and Yuepu Pu

Table S1. The concentrations of MC-LR for different samples with different incubation time (mg/L).

Time / h	Control	G0	G1	G2	G3
0	1.00 ± 0.03	1.00 ± 0.04	1.00 ± 0.02	1.00 ± 0.03	1.00 ± 0.01
12	1.07 ± 0.05	0.89 ± 0.05	0.79 ± 0.03	0.70 ± 0.06	0.65 ± 0.03
24	0.99 ± 0.03	0.87 ± 0.03	0.73 ± 0.03	0.58 ± 0.05	0.51 ± 0.04
36	1.05 ± 0.04	0.85 ± 0.07	0.70 ± 0.07	0.50 ± 0.04	0.37 ± 0.04
48	0.92 ± 0.04	0.74 ± 0.05	0.57 ± 0.04	0.34 ± 0.02	0.22 ± 0.04
60	0.94 ± 0.04	0.74 ± 0.04	0.42 ± 0.06	0.27 ± 0.04	0.14 ± 0.03
72	0.86 ± 0.04	0.66 ± 0.05	0.37 ± 0.04	0.15 ± 0.03	0.08 ± 0.02
84	0.93 ± 0.07	0.54 ± 0.04	0.24 ± 0.02	0.11 ± 0.03	0.04 ± 0.01
96	0.92 ± 0.05	0.48 ± 0.04	0.19 ± 0.06	0.05 ± 0.02	0.00 ± 0.00
108	0.97 ± 0.04	0.36 ± 0.05	0.16 ± 0.05	0.00 ± 0.00	0.00 ± 0.00
120	0.95 ± 0.05	0.26 ± 0.08	0.08 ± 0.03	0.00 ± 0.00	0.00 ± 0.00
132	0.97 ± 0.04	0.21 ± 0.04	0.03 ± 0.02	0.00 ± 0.00	0.00 ± 0.00
144	1.01 ± 0.05	0.18 ± 0.03	0.00 ± 0.00	0.00 ± 0.00	0.00 ± 0.00
156	0.94 ± 0.05	0.16 ± 0.03	0.00 ± 0.00	0.00 ± 0.00	0.00 ± 0.00
168	0.95 ± 0.04	0.13 ± 0.03	0.00 ± 0.00	0.00 ± 0.00	0.00 ± 0.00

Data are shown as mean values ± standard deviation (SD) (n = 3).

Table S2. Utilization of various carbon sources by the sediment bacterial communities in different samples after 168 h of incubation.

Samples	Carbohydrates	Amino Acids	Polymers	Phenolic Compounds	Amines	Carboxylic Acids
G0	0.71 ± 0.04	0.84 ± 0.09	1.03 ± 0.10	0.47 ± 0.07	0.80 ± 0.16	0.66 ± 0.09
G1	0.67 ± 0.07	0.36 ± 0.04*	0.70 ± 0.01	0.28 ± 0.05	0.59 ± 0.04	0.36 ± 0.05
G2	0.37 ± 0.17	0.23 ± 0.13*	0.45 ± 0.22	0.19 ± 0.17	0.34 ± 0.08	0.33 ± 0.06
G3	0.18 ± 0.04	0.30 ± 0.05*	0.39 ± 0.09	0.08 ± 0.07*	0.22 ± 0.02	0.32 ± 0.05

Data are shown as mean values ± SD (n = 3), * $p < 0.05$ (sample G0 versus MC-LR treated samples).
